# Switching of mesodermal and endodermal properties in hTERT-modified and expanded fetal human pancreatic progenitor cells

**DOI:** 10.1186/scrt6

**Published:** 2010-03-15

**Authors:** Kang Cheng, Antonia Follenzi, Manju Surana, Norman Fleischer, Sanjeev Gupta

**Affiliations:** 1 Hepatology Division, Department of Medicine, Albert Einstein College of Medicine, Ullmann Bldg., Rm 625, 1300 Morris Park Avenue, Bronx, NY 10461, USA; 2 Department of Pathology, Albert Einstein College of Medicine, Ullmann Bldg., Rm 625, 1300 Morris Park Avenue, Bronx, NY 10461, USA; 3 Endocrinology Division, Department of Medicine, Diabetes Research Center, Albert Einstein College of Medicine, Forchheimer Bldg., Rm 505, 1300 Morris Park Avenue, Bronx, NY 10461, USA; 4 Hepatology Division, Department of Medicine, Cancer Research Center, Diabetes Research Center, Center for Human Embryonic Stem Cell Research, Marion Bessin Liver Research Center, Ruth L. and David S. Gottesman Institute for Stem Cell and Regenerative Medicine Research, and Institute for Clinical and Translational Research, Albert Einstein College of Medicine, Ullmann Bldg., Rm 625, 1300 Morris Park Avenue, Bronx, NY 10461, USA

## Abstract

**Introduction:**

The ability to expand organ-specific stem/progenitor cells is critical for translational applications, although uncertainties often arise in identifying the lineage of expanded cells. Therefore, superior insights into lineage maintenance mechanisms will be helpful for cell/gene therapy.

**Methods:**

We studied epithelial cells isolated from fetal human pancreas to assess their proliferation potential, changes in lineage markers during culture, and capacity for generating insulin-expressing beta cells. Cells were isolated by immunomagnetic sorting for epithelial cell adhesion molecule (EpCAM), and characterized for islet-associated transcription factors, hormones, and ductal markers. Further studies were performed after modification of cells with the catalytic subunit of human telomerase reverse transcriptase (hTERT).

**Results:**

Fetal pancreatic progenitor cells efficiently formed primary cultures, although their replication capacity was limited. This was overcome by introduction and expression of hTERT with a retroviral vector, which greatly enhanced cellular replication *in vitro*. However, we found that during culture hTERT-modified pancreatic progenitor cells switched their phenotype with gain of additional mesodermal properties. This phenotypic switching was inhibited when a pancreas-duodenal homeobox (Pdx)-1 transgene was expressed in hTERT-modified cells with a lentiviral vector, along with inductive signaling through activin A and serum deprivation. This restored endocrine properties of hTERT-modified cells *in vitro*. Moreover, transplantation studies in immunodeficient mice verified the capacity of these cells for expressing insulin *in vivo*.

**Conclusions:**

Limited replication capacity of pancreatic endocrine progenitor cells was overcome by the hTERT mechanism, which should facilitate further studies of such cells, although mechanisms regulating switches between meso-endodermal fates of expanded cells will need to be controlled for developing specific applications. The availability of hTERT-expanded fetal pancreatic endocrine progenitor cells will be helpful for studying and recapitulating stage-specific beta lineage advancement in pluripotent stem cells.

## Introduction

Cell therapy for diabetes mellitus will be advanced by the availability of additional donor cells. Islet cells may originate from pancreatic ductal or other epithelial cells, especially after damage in the adult pancreas [1,2]. However, the replication potential of human pancreatic islet cells is limited, which generally restricts expansion of these cells under culture conditions [3]. Although cell lines have been generated by oncogenetic transformation of islet cells [4,5] tumorigenic cells will obviously be unsuitable for basic studies under various contexts, as well as for cell therapy. This problem of genetic transformation afflicts other sources of cells, for example, inducible pluripotential stem cells (iPS), which are teratogenic [6]. Whereas reprogramming of cells through transcription factor modifications was recently found to be effective for transdifferentiating pancreatic exocrine cells to endocrine beta cells in vivo [7], generalization of this observation in developing treatments for diabetes mellitus requires much more work.

We consider fetal tissues to be of considerable interest as cell donors because these are particularly enriched in lineage-committed stem/progenitor cells. However, in comparison with pluripotent human embryonic stem cells (hESC) or iPS, the replication potential of fetal cells was limited [8,9], possibly due to telomere shortening [3,10]. Modification of fetal human liver cells with the catalytic subunit of human telomerase reverse transcriptase (hTERT) enhanced cell replication without loss of stem/progenitor cell properties and additional expression of pancreatic duodenal homeobox (Pdx)-1 in these cells induced regulated insulin expression [10,11]. On the other hand, we recently determined that epithelial fetal human liver cells became altered under continuous culture, with development of a novel conjoint meso-endodermal phenotype under control of transcriptional regulation mechanisms [9]. As nuclear transcription factors are of fundamental significance in directing embryonic development of the foregut endoderm, which originates both hepatic and pancreatic stem/progenitor cells [12], we considered that pancreatic fetal stem/progenitor cells may share this property of lineage switching.

Among lineage-specific mechanisms, some transcription factors are of particular significance in pancreatic lineage development, for example, Pdx1, Neurogenin 3 (NGN3), and others, while cytokine networks represent another level of endocrine regulation in stem cells, for example, activin A - a member of the transforming growth factor (TGF)-β superfamily - inducts beta cell differentiation in hESC [2,12,13].

To generate further insights into fetal human endocrine stem/progenitor cells, we isolated epithelial cells characterized by the display of epithelial cell adhesion molecule (EpCAM), and studied mechanisms of proliferation and differentiation. This led us to define phenotypic changes in cells during culture, including after increased cell replication with hTERT expression. We found that the initial epithelial/endodermal phenotype of fetal endocrine cells was altered with gain of mesenchymal/mesodermal phenotype, which was similar to changes in stem/progenitor cells isolated from fetal human livers, further emphasizing sharing of mechanisms in cells originating from the foregut endoderm [9]. The ability to control the phenotype of these cells through additional manipulations offers potential ways to regulate cell differentiation for superior β cell functions and to understand mechanisms in stage-specific β lineage advancement in other types of stem/progenitor cells.

## Materials and methods

### Human UHHtissues

Fetal tissues of 17 to 24 week gestation were from Human Fetal Tissue Repository of Albert Einstein College of Medicine (Bronx, NY, USA). A total of 20 fetal pancreata were used. Mature pancreatic islets were from Islet Distribution Program of Juvenile Diabetes Research Foundation New York, NY, USA. Procedures for fetal tissue collection, including informed consent in writing from donors, as well as this research were approved by the Committee on Clinical Investigations (Institutional Review Board) at Einstein.

### Cell isolation and culture

Fetal pancreases were rinsed in Leffert's buffer (10 mM HEPES, 3 mM KCl, 130 mM NaCl, 1 mM NaH_2_PO_4 _and 10 mM glucose, pH 7.4) with 5 mM CaCl_2_, 100 U/ml DNase (Worthington Biochemical Corp., Lakewood, NJ, USA), and 0.03% collagenase P (Roche Applied Science, Indianapolis, IN, USA). Tissue was repeatedly passed through 5 ml syringe and gently agitated in this buffer for 20 to 30 minutes at 37°C. Dissociated cells were passed through 80 μm dacron (Sefar Filtration Inc. Depew, NY, USA) and washed twice in phosphate buffered saline (PBS, pH 7.2), 2 mM EDTA, 0.5% BSA (wash buffer) under 800 × g for five minutes at 4°C. After resuspending 5 × 10^7 ^cells in 0.3 ml wash buffer, FcR blocking reagent was added, and cells were incubated with 100 μl microbeads conjugated with antibody against human EpCAM (Miltenyi Biotec Inc, Bergisch Gladbach, Germany) for 30 minutes at 4°C. To verify cell separation, aliquots were incubated with FITC-conjugated anti-EpCAM (Miltenyi Biotec) for 10 minutes at room temperature and examined under epifluorescence. Remaining cells were resuspended in 1 ml PBS with pelleting of EpCAM-positive and -negative fractions in wash buffer under 300 × g for 10 minutes. Cell viability was determined by exclusion of 0.2% trypan blue dye.

For culture, 4 × 10^3 ^cells were plated in 35 mm dishes in Dulbecco's Minimal Eagle Medium (DMEM) (Life Technologies Inc., Rockville, MD, USA) with 2.5 mM glucose, 100 U/ml penicillin, 100 μg/ml streptomycin, 250 ng/ml amphotericin B, and 10% fetal bovine serum (FBS, Atlanta Biologicals Inc., Norcross, GA, USA) in 5% CO_2 _at 37°C. The medium was replaced twice weekly and near-confluent cells were subpassaged 1:3 with trypsin-EDTA for three minutes at 37°C. We analyzed primary cells after short-term (1 to 2 d) or long-term (10 to 14 d) culture, followed by serial passages until cell replication declined. Cells expressing hTERT were cultured for >50 passages. Changes in cell population doublings were determined during culture by manual counting of cell numbers. To induce insulin expression, cells were cultured in serum-free medium for seven days and with 4 nM human recombinant activin A (R&D Systems, Minneapolis, MN, USA) for another three days.

### hTERT retrovirus

The vector encoding hTERT and puromycin selection marker was previously described [10]. EpCAM-positive cells were transduced with 2 to 4 × 10^4 ^units/ml of hTERT retrovirus. Fresh DMEM was added after 24 h and cells were cultured to 70-80% confluency before adding 0.75 μM puromycin (Sigma Biochemical Co., St. Louis, MO, USA). A clone of EpCAM-positive cells transduced with hTERT was designated hTERT-FPC.

### Pdx1 lentivirus vector (LV)

The pONY4-Pdx1 plasmid containing rat Pdx1 cDNA was provided by Dr. S. Efrat (Tel Aviv University, Tel Aviv, Israel). Pdx1 cDNA was excised by BamHI and SalI and 850 base pair fragment was subcloned into pCCLsinPPT.hPGK.IRES.GFP.Wpre plasmid to obtain transfer plasmid designated pCCLsinPPT.hPGK.Pdx1.IRES.GFP.Wpre. Pdx1-LV was produced by calcium phosphate transfection of 293T cells as described [14]. pMDLg/pRRE was the HIV-derived packaging construct, pRSV-Rev construct expressed Rev regulatory protein and pMD2.G construct expressed VSV-G envelope protein. To obtain high-titer LV, 293T medium was concentrated under 50,000 × g for 140 minutes with tittering of LV in HeLa cells. To transduce cells, LV under 10 multiplicities of infection (MOI) was added to cells overnight at 37°C. Cell transduction was analyzed after 72 h with flow cytometry for green fluorescent protein (GFP).

### Cytostainings

A total of 4 × 10^3 ^cells were cultured on glass cover slips and fixed in cold ethanol for 10 minutes. Histochemical staining for glycogen, dipeptidyl peptidase IV (DPPIV) and γ-glutamyl transpeptidase (GGT) was as previously described [9]. For immunostaining, cells were fixed in 4% paraformaldehyde, blocked in goat or donkey serum with 0.1% Triton X-100 in PBS for 10 minutes, and incubated for one hour with antibodies at room temperature (Table 1). To measure intensity of immunostaining, microphotographs were analyzed with ImageJ software (NIH, Bethesda, MD, USA).

**Table 1:** Immunostaining protocols

Antigen	Blocking	Primary Antibody	Dilution	Secondary Antibody	Dilution
**Insulin**	3% Goat serum	AB3440, Guinea Pig anti-human insulin (Chemicon El Segundo, CA)	1:100	Rhodamine-conjugated anti-guinea pig IgG (AB7136, Abcam, Cambridge, MA)	1:500
**Glucagon**	3% Goat serum	AB932, Rabbit anti-Glucagon (Chemicon El Segundo, CA)	1:100	Goat anti-Rabbit IgG, Cy3-conjugated (AP132C, Chemicon)	1:500
**C-peptide**	3% Goat serum	ab14181, Rabbit anti-c-peptide (Abcam Inc, Cambridge, MA)	1:100	Goat anti-Rabbit IgG, Cy3-conjugated (AP132C, Chemicon)	1:500
**Vimentin**	5% Donkey serum	V212210, (United States Biologicals, Swampscott, MA)	1:100	Rhodamine-conjugated anti mouse IgG (#715-295-150, Jackson Immuno Research)	1:500

IgG, Immunoglobulin G.

### Insulin radioimmunoassay

Cells were lysed for measuring insulin and c-peptide as previously described [4]. Data were normalized to cell numbers.

### Reverse-transcription polymerase chain reaction (RT-PCR)

Total RNA was extracted with Trizol Reagent (Invitrogen Life Technologies, Carlsbad, CA, USA). After incubation with Amplification Grade DNase I (Invitrogen), RNAs were reverse transcribed with Omniscript RT system (Qiagen Inc., Valencia, CA, USA). Equal amounts of cDNAs were amplified with Platinum PCR Supermix (Invitrogen) as follows: 94°C × 5 minutes and 35 cycles at 94°C × 30 sec, annealing at various temperatures for 30 sec, and 72°C for one minute, with final extension at 72°C for seven minutes. PCR products were resolved in 1.5% agarose containing ethidium bromide. Oligonucleotide primers, annealing conditions and expected product sizes are listed in Table 2, including previously published conditions [9-11,15-17].

**Table 2:** RT-PCR Primers

Gene	Primer sequences (5'-3'): Forward and Reverse	Tm (°C)	Product (basepairs)	References
Insulin	GCTGCATCAGAAGAGGCCATCAGGC GCGTCTAGTTGCAGTAGTTCTCCAG	58	380	[11]
Glucagon	GAATTCATTGCTTGGCTGGTGAAAGGC CATTTCAAACATCCCACGTGGCATGCA	60	255	[11]
Pancreatic Polypeptide	CTGCTGCTGCTGTCCACCTGCGTG CTCCGAGAAGGCCAGCGTGTCCTC	60	206	[11]
Somatostatin	CGTCAGTTTCTGCAGAAGTCCCTGGCT CCATAGCCGGGTTTGAGTTAGCAGATC	60	206	[11]
NeuroD	ATCCCAACCCACCACCAACC CAGCGGTGCCTGAGAAGATT	60	440	[15]
Pancreatic duodenal homeobox1 gene (Pdx1)	CTGCCTTTCCCATGGATGAA CGCTTCTTGTCCTCCTCCTTT	58	277	[15]
Prohormone convertase (PC1/3)	TTGGCTGAAAGAGAACGGGATACATCT ACTTCTTTGGTCATTGCTTTGGCGGTG	65	456	[11]
Prohormone convertase (PC2)	GCATCAAGCACAGACCTACACTGG GAGACACAAACCACCCTTCATCCTTC	60	308	[11]
Chromogranin-A	CGGACAGTTCCATGAAGCTCTC GAGTCAGGAGTAGGAGACAAGG	58	444	[11]
Glucokinase	GACGAGTTCCTGCTGGAGTATGAC GACTCGATGAAGGTGATCTCGCAGCTG	65	523	[11]
Paired homeobox gene4 (PAX4)	CACCTCTCTGCCTGAGGACACGGTGAG CTGCCTCATTCCAAGCCATACAGTAGTG	64	443	[11]
Paired homeobox gene 6 (PAX6)	CAGTCACAGCGGAGTGAATCAGC GCCATCTTGCGTAGGTTGCCCTG	58	519	[11]
Glucose transporter2 (GLUT2)	GCCATCCTTCAGTCTCTGCTACTC GCTATCATGCTCACATAACTCATCCA	65	523	[11]
Betacellulin2 (BETA2)	CCTGAGCAGAACCAGGACATGCC ATCAAAGGAAGGGCTGGTGCAATCA	58	221	[11]
Neurogenin3 (NGN3)	ACTGAGCAAGCAGCGACGGAGTC GCACCCACAGCCGAGCGACAGAC	65	448	[11]
NK homeobox protein 6.1 (NKX6 1)	CTCCTCCTCGTCCTCGTCGTCGTC CTTGACCTGACTCTCTGTCATC	60	699	[11]
NK homeobox protein 2.2 (NKX2 2)	CGGACAATGACAAGGAGACCCCG CGCTCACCAAGTCCACTGCTGCTGG	65	490	[11]
Islet1 (ISL1)	GTGCGGAGTGTAATCAGTATTTGG GTCATCTCTACCAGTTGCTCCTTC	58	519	[11]
GATA2 transcription factor	CGTCTTCTTCAATCACCTCG CGTCTTGGAGAAGGGGCTC	55	225	[10]
GATA6 transcription factor	GAAGAAGCACATGATTTTGGAC GATGGAAGGGAAGGGCCAG	58	181	[10]
Transforming growth factor alpha (TGFα)	ATGGTCCCCTCGGCTGGA GGCCTGCTTCTTCTGGCTGGCA	58	297	[10]
Transforming growth factor beta1 (TGFβ1)	GCCCTGGACACCAACTATTGCT AGGCTCCAAATGTAGGGGCAGG	58	161	[10]
Transforming growth factor beta2 (TGFβ2)	GATTTCCATCTACAAGACCACGAGGGACTTGC CAGCATCAGTTACATCGAAGGAGAGCCATTCG	65	503	[10]
Transforming growth factor beta1 receptor (TGFβ1R)	CGTGCTGACATCTATGCAAT AGCTGCTCCATTGGCATAC	54	251	[10]
Transforming growth factor beta2 receptor (TGFβ2R)	TGCACATCGTCCTGTGGAC GTCTCAAACTGCTCTGAAGTGTTC	58	784	[10]
Insulin-like growth factor receptor (IGFR)	ACCCGGAGTACTTCAGCGCT CACAGAAGCTTCGTTGAGAA	55	229	[10]
Cytokeratin-19	ATGGCCGAGCAGAACCGGAA CCATGAGCCGCTGGTACTCC	60	308	[16]
α-Smooth muscle actin	AGTACCCGATAGAACATGG TTTTCTCCCGGTTGGC	60	153	[9]
Vimentin	CACCTACAGCCTCTACG AGCGGTCATTCAGCTC	60	170	[9]
Human telomerase reverse transcriptase (hTERT)	AAGTTCCTGCACTGGCTGATGAG TCGTAGTTGAGCACGCTGAACAG	60	378	[17]
β-actin	AGAGCTATGAGCTGCCTGAC CTGATCCACATCTGCTGGAA	55	361	[15]
Glyceraldehyde phosphate dehydrogenase (GAPDH)	CCATGGAGAAGGCTGGGG CAAAGTTGTCATGGATGACC	58	194	[10]

### Cell transplantation studies

The Animal Care and Use Committee of Albert Einstein College of Medicine approved animal use in conformity with National Research Council's *Guide for the Care and Use of Laboratory Animals *(United States Public Health Service publication, revised 1996). All animals were maintained in the Institute of Animal Studies at Albert Einstein College of Medicine. We transplanted 2 × 10^6 ^hTERT-FPC modified by Pdx1 LV in NOD-CB17-prkdc-SCID mice (Jackson Labs., Bar Harbor, ME, USA) into portal vein as described previously [18]. Animals were sacrificed in groups (n = 3 each) 24 hours and one, two or four weeks after transplantation to analyze cell engraftment and gene expression. To verify presence of transplanted cells in the liver, genomic DNA was isolated by Trizol Reagent. A primate-specific sequence from the Charcot-Marie-Tooth (CMT)-1A element was amplified by PCR and in situ hybridization was performed with a pancentromere probe to identify human cells as described [19]. GFP immunostaining was performed as described [18]. In some studies, tissues were additionally stained for insulin as described above. After washing with PBS, tissues were counterstained with DAPI (4',6-diamidino-2-phenylindole)-antifade (Molecular Probes, Invitrogen Life Technologies Corporation, Carlsbad, CA, USA) and examined under epifluorescence.

### Statistical analysis

Data are shown as means ± SD. Significances were examined by t-test. *P *values < 0.05 were considered significant.

## Results

### Characterization of pancreatic epithelial cells

**Figure 1:**
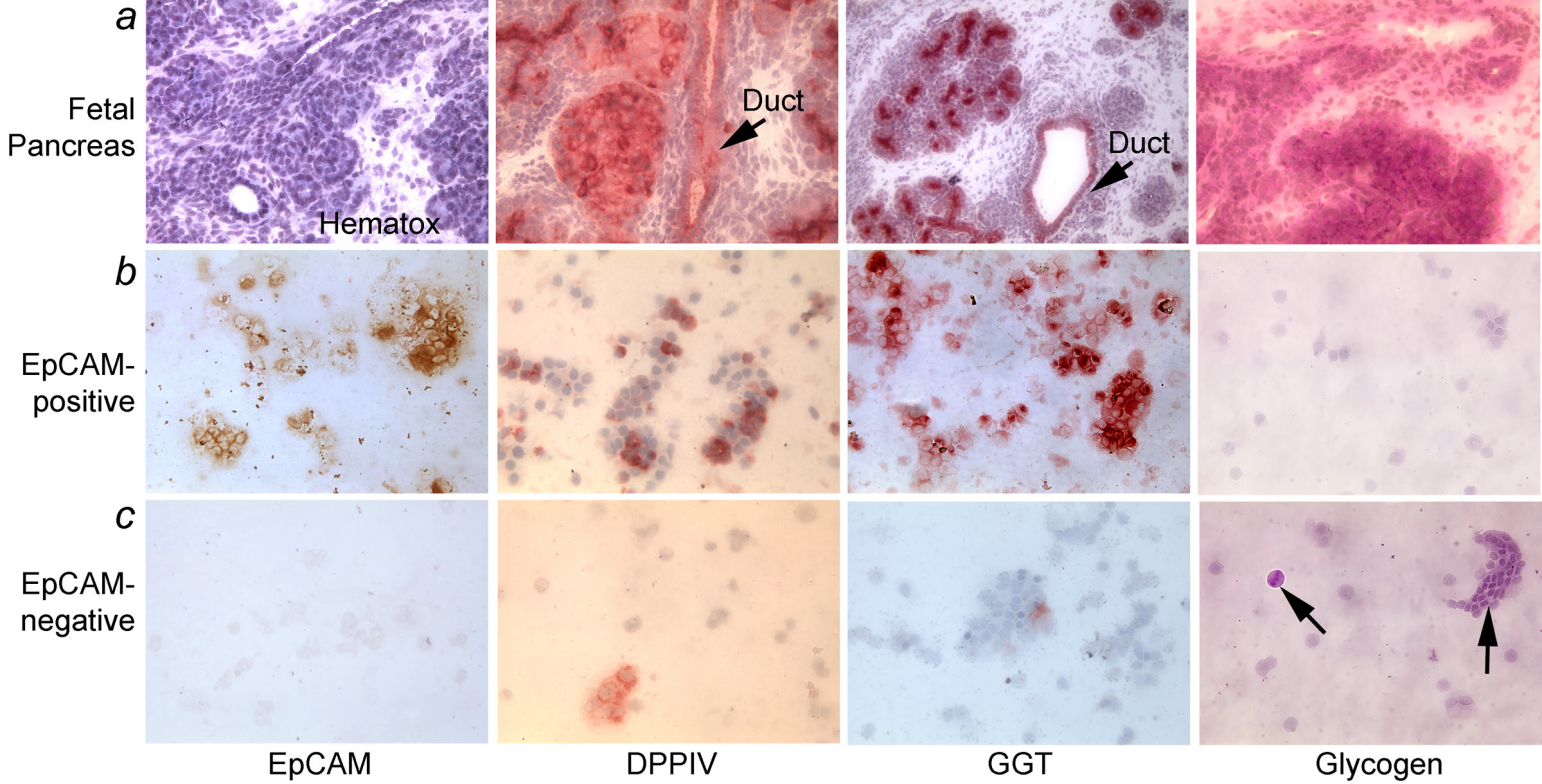
**Fractionation of EpCAM-positive epithelial cells from fetal human pancreas**. **(a) **shows intact 22-week fetal human pancreas with hematoxylin staining alone (extreme left) or histochemical staining for DPPIV, GGT and glycogen, as indicated, which were expressed in cells in ductal (arrows), periductal and acinar regions. **(b) **shows EpCAM-positive cells isolated by immunomagnetic cell sorting with EpCAM, DPPIV and GGT expression but absence of glycogen. **(c) **shows EpCAM-negative cell fraction with only occasional epithelial cells and more abundant glycogen-containing acinar cells (arrows, panel extreme right). Orig. mag., a, ×200; b and c, ×400.

After immunomagnetic sorting, we obtained 1-3 × 10^7 ^EpCAM-positive epithelial cells per fetal pancreas with viability of 85 to 90%. Intact fetal pancreas showed primitive acini, as well as islets with ductal and vascular structures (Figure 1a). Cells in ductal, periductal and acinar regions expressed the ductal markers, DPPIV and GGT. EpCAM staining verified the presence of epithelial cells with DPPIV and GGT expression in those areas. Similarly, EpCAM-positive isolated cells expressed DPPIV and GGT, whereas cells with glycogen, which is a characteristic of pancreatic acinar cells and not beta cells, were not observed in this fraction (Figure 1b). By contrast, the EpCAM-negative cell fraction showed only infrequent EpCAM, DPPIV or GGT-positive cells, whereas cells with glycogen were frequent (Figure 1c).

**Figure 2:**
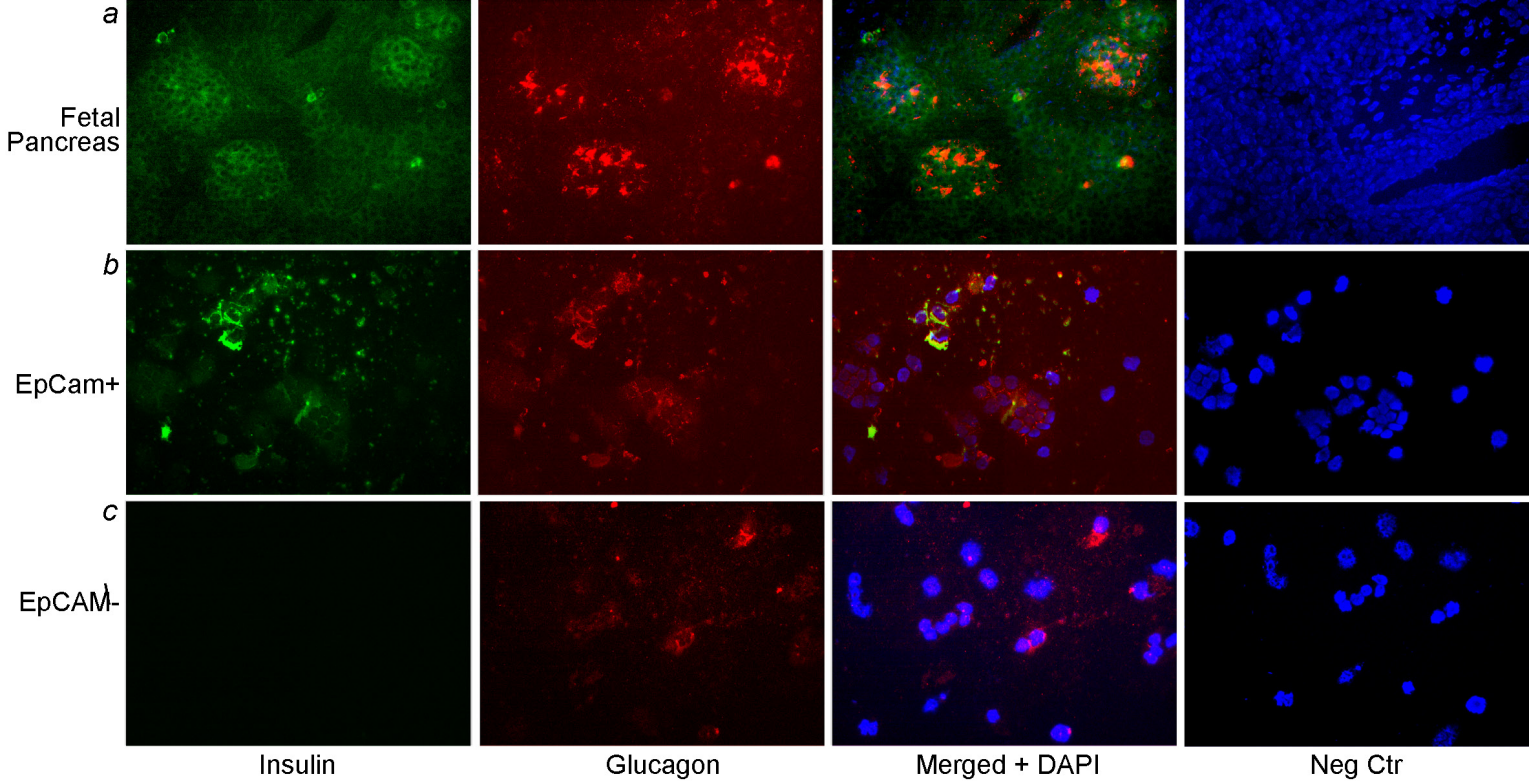
**Endocrine phenotype of EpCAM-positive fetal pancreatic cells**. **(a) **shows immunostaining of 22-week fetal human pancreas to demonstrate cells in primitive pancreatic islets with coexpression of insulin and glucagon. Negative controls, where primary antibodies were omitted, are on extreme right. **(b) **shows isolated freshly EpCAM-positive cells with coexpression of insulin and glucagon in some cells. **(c) **shows EpCAM-negative fraction showing occasional cells with glucagon. Orig. mag., a, ×200; b and c, ×400.

Cells in the EpCAM-positive fraction expressed insulin or glucagon, often both, whereas in the EpCAM-negative fraction, cells expressing insulin or glucagon were rare (Figure 2). Taken together, these findings indicated that EpCAM selected epithelial cells and that endocrine progenitor cells were present within epithelial cell niches in the fetal pancreas.

**Figure 3:**
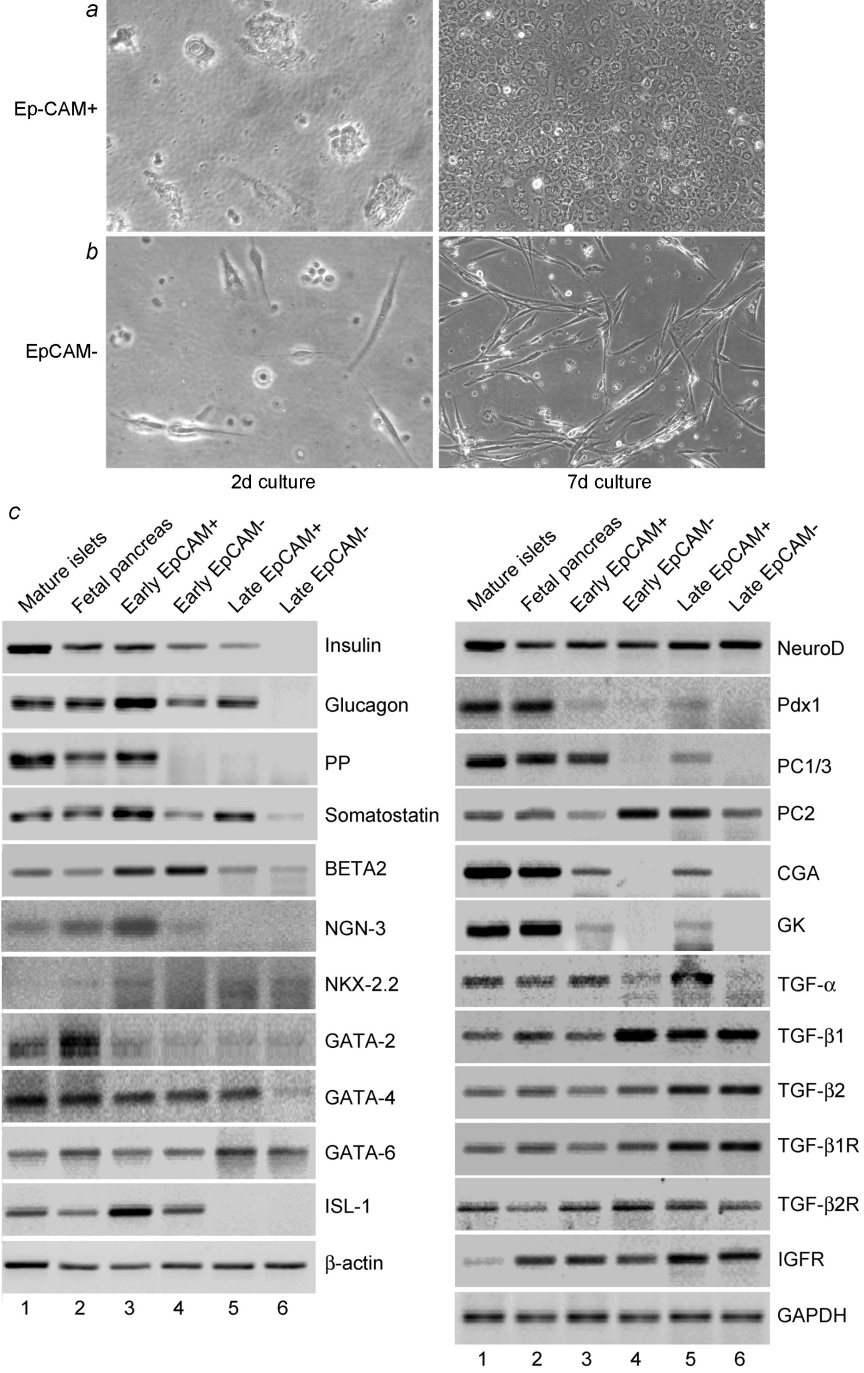
**Initial characterization of fetal pancreatic cells**. **(a) **and **(b) **show morphology of cells in culture after 2 d and 7 d. Note epithelial morphology of EpCAM-positive cells. **(c) **shows RT-PCR for genes as indicated. Lanes 1 to 6 show results from mature human pancreatic islets, intact fetal pancreas, cells after early term culture (1 to 2 d) or longer culture (10 to 14 d). For comparisons, β-actin and glyceraldehyde phosphate dehydrogenase (GAPDH) genes were included.

After culture, differences in EpCAM-positive and -negative fractions were apparent, as the former retained epithelial morphology, whereas the latter showed spindle-shaped morphology (Figure 3a and 3b). In primary (P0) culture, EpCAM-positive cells proliferated within several days to form confluent monolayers of cells, although replication of cells declined over 10 to 12 passages, and cells required greater times to form confluent cultures. It was noteworthy that cells continued to express EpCAM, as shown by immunostaining, which simultaneously verified the absence of nonepithelial cells in cultures. Also, EpCAM-positive cells expressed pancreatic polypeptide (PP), somatostatin, glucagon and insulin in short-term culture (1 to 2 d), whereas after longer-term culture (10-14 d), PP was no longer expressed (Figure 3C). By contrast, in EpCAM-negative cells, insulin, glucagon and somatostatin were expressed at lower levels and only in the short-term, while PP was not expressed at all. Pdx1 was expressed in EpCAM-positive cells in short- and long-term cultures. BETA2 and NeuroD transcription factors, which regulate β-cell differentiation, were expressed in EpCAM-positive cells, as were transcriptional regulators, GATA-2, GATA-4 and GATA-6, which govern differentiation of additional mesodermal lineages. NGN3 was expressed in EpCAM-positive cells. Other regulatory transcription factors, for example, Pax-4, Pax-6, Nkx-2.2 and Nkx-6.1, were either not expressed, or were expressed less well in EpCAM-positive cells, although Isl-1, which promotes islet cell development, was expressed during short-term cell culture. The glucose transporter, GLUT-2, and glucokinase (GK), which participate in signal-secretion coupling in β-cells, were expressed in only EpCAM-positive cells. These cells expressed proinsulin processing enzymes, prohormone convertases, PC1/3 and PC2, and chromogranin A (CGA), the major component of dense-core secretory granules. Finally, growth factor genes regulating islet and β-cell development, for example, TGF-α, TGF-β1, TGF-β2, and their receptors, as well as insulin-like growth factor-1 receptor (IGF-1R), were expressed.

### Proliferation of EpCAM-positive cells after telomerase reconstitution

**Figure 4:**
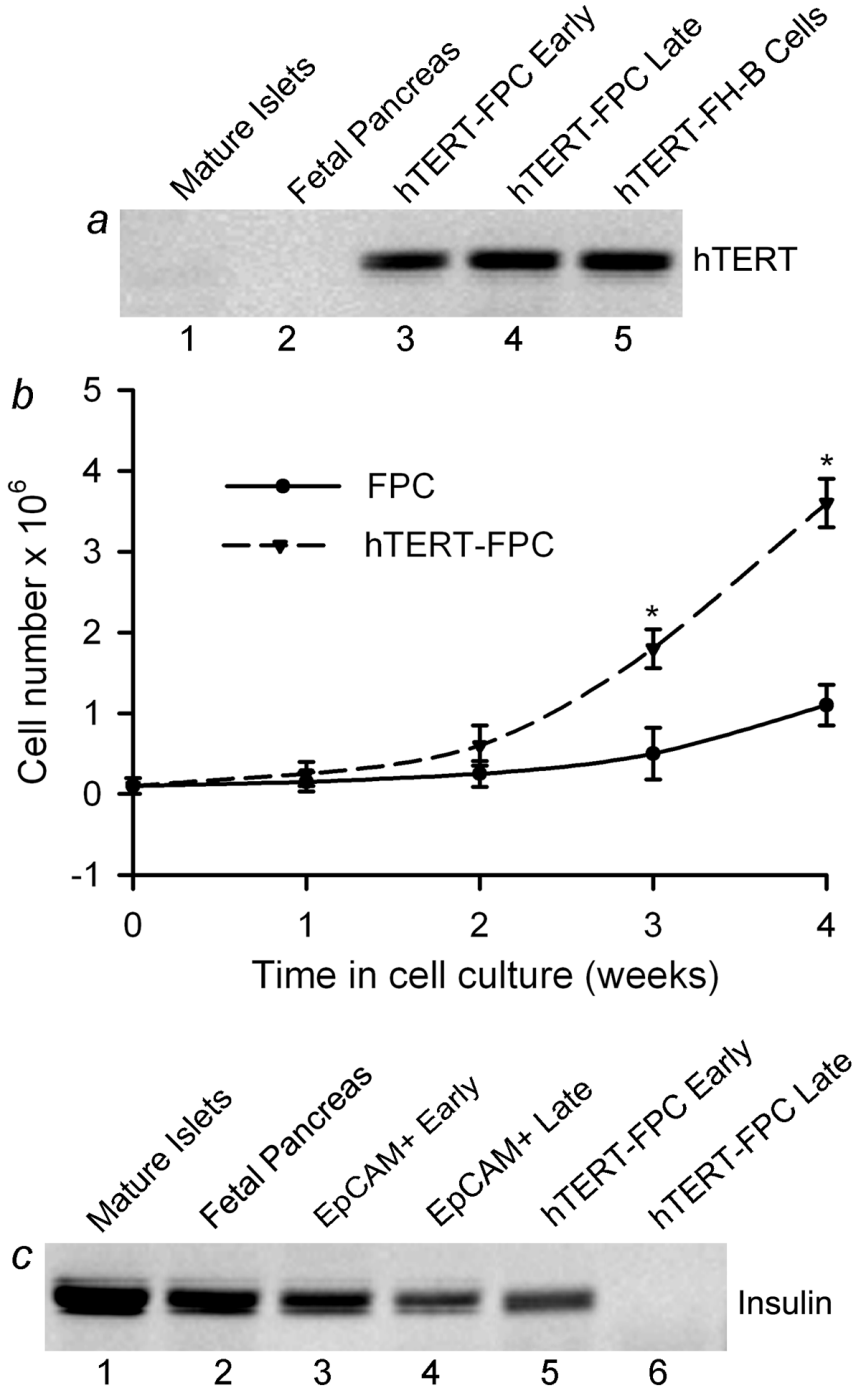
**Immortalization of EpCAM-positive fetal pancreatic cells by hTERT**. **(a) **shows RT-PCR for hTERT expression after early and late passages of hTERT-FPC. Note the absence of hTERT expression in mature human islets and fetal pancreas, whereas hTERT-FH-B fetal human liver cells expressed hTERT (positive control). **(b) **shows kinetics of proliferation in primary EpCAM-positive fetal pancreatic cells and hTERT-FPC during culture over up to four weeks, which was 4-6-fold greater in the latter. Asterisks indicate *P *< 0.05. **(c) **shows RT-PCR for insulin, which was expressed in mature islets (lane 1), fetal pancreas (lane 2) and primary EpCAM-positive cells during early and late culture (lanes 3 and 4), as well as an early passage (P3) of hTERT-FPC (lane 5) but not in hTERT-FPC after further cell culture (lane 6).

In mature pancreatic islets or in fetal pancreas, hTERT mRNA was absent (Figure 4a). After hTERT introduction, RT-PCR showed hTERT mRNA in transduced cells, similar to hTERT-FH-B fetal human liver cells [10]. Serial cultures of hTERT-FPC demonstrated robust proliferation despite >50 passages, during which >170 estimated cell population doublings occurred, which was beyond the boundaries of replicative senescence in somatic cells. Comparison of cell doubling rates showed proliferation was significantly increased in FPC after expression of hTERT (Figure 4b). The human identity of hTERT-FPC was verified by PCR of DNA with human-specific CMT-1A probe, as well as in situ hybridization for primate-specific alphoid satellite sequences in centromeres [19]. Morphologically, hTERT-FPC resembled unmanipulated primary EpCAM-positive fetal cells. To address whether hTERT-FPC maintained endocrine functions, we determined insulin expression (Figure 4c). Primary EpCAM-positive fetal cells expressed insulin during culture over up to two weeks and insulin was expressed in cultured hTERT-FPC after up to four passages (P4). Subsequently, insulin expression declined in hTERT-FPC, and was undetectable after 10 passages. This was associated with a parallel decline, and eventual loss, of Pdx1 expression in hTERT-FPC, indicating that the β-cell phenotype of hTERT-FPC was perturbed during cell culture.

### Restoration of endocrine phenotype in hTERT-FPC

**Figure 5:**
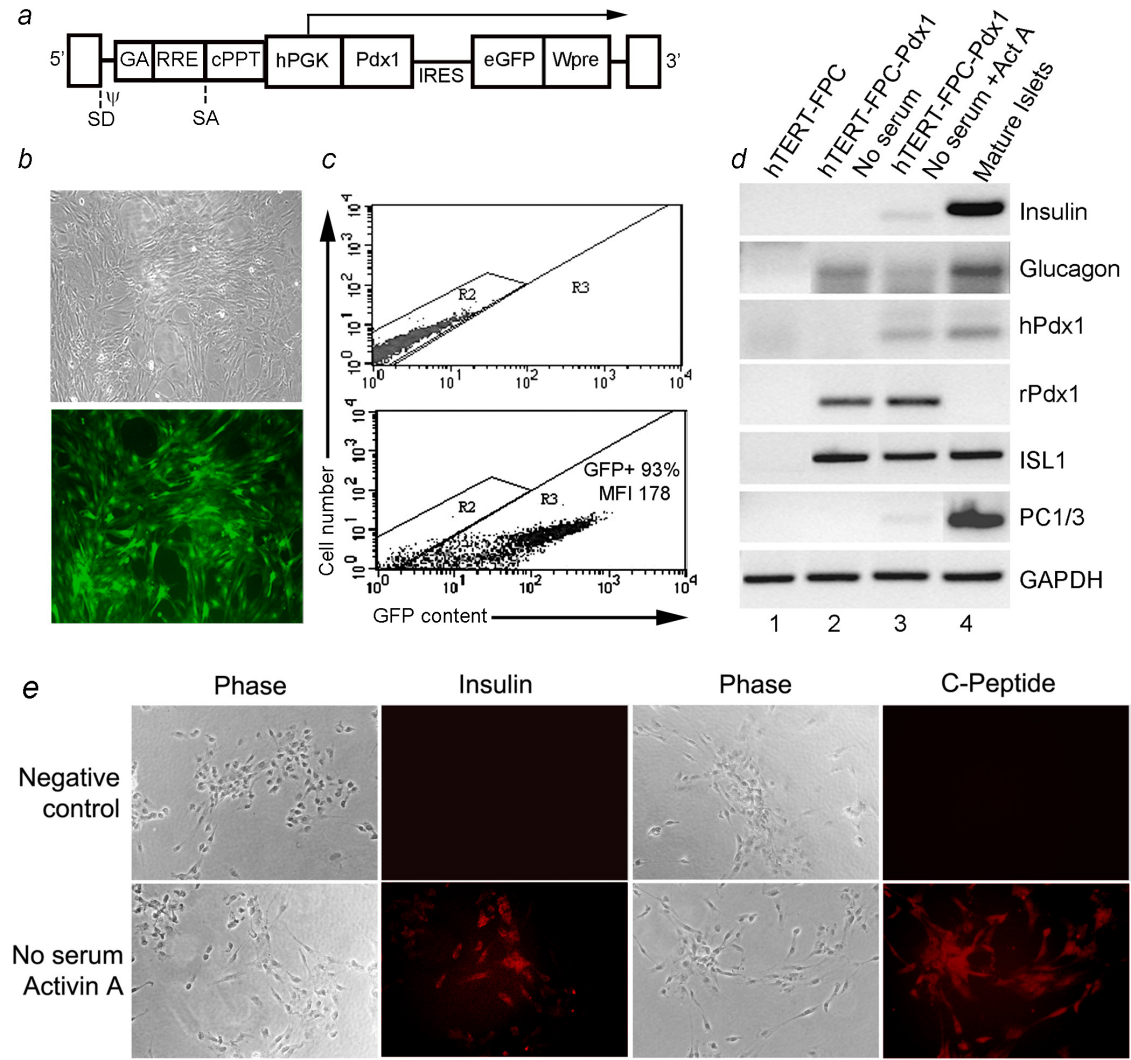
**Induction of insulin-expression in hTERT-FPC by Pdx1-LV**. **(a) **shows schematic of LV with rat Pdx1 and GFP genes driven by hPGK promoter - IRES, intervening internal ribosomal entry site, cPPT, central polypurine tract, Wpre, posttranscriptional regulatory element of the woodchuck hepadnavirus. **(b) **shows Pdx1-LV-transduced hTERT-FPC under phase contrast (top) and under epifluorescence for GFP. **(c) **shows flow cytometric quantitation of GFP in nontransduced cells (top panel) and Pdx1-LV-transduced hTERT-FPC. MFI = mean fluorescence intensity. **(d) **shows RT-PCR for gene expression in control hTERT-FPC (lane 1), Pdx1-LV-transduced hTERT-FPC cultured without serum (lane 2) and without serum plus activin A (lane 3), and mature pancreatic islets (lane 4). **(e) **shows insulin and c-peptide expression in negative control hTERT-FPC-Pdx1 cells, where primary antibodies were omitted, and cells with expression of both insulin and c-peptide. Orig. Mag., × 200.

We studied whether restoring Pdx1 expression will reconstitute the endocrine phenotype of hTERT-FPC. Therefore, we expressed rat Pdx1 and green fluorescent protein (GFP) under phosphogylcerate kinase (PGK) promoter, which is ubiquitously active (Figure 5a). After transduction of hTERT-FPC with Pdx1 LV >90% cells were GFP-positive (Figure 5b and 5c). GFP expression was maintained over multiple cell passages. Rat Pdx1 induced glucagon and ISL1 expression in hTERT-FPC, without expression of endogenous hPdx1 or insulin under basal conditions (Figure 5d). However, when hTERT-FPC were cultured in serum-free medium with activin A, hPdx1 and insulin mRNA were expressed and hTERT-FPC-Pdx1 also contained immunostainable insulin and c-peptide. Neither hPdx1 nor insulin was detected in hTERT-FPC without transduction with Pdx1 LV. No insulin was detected by immunoassay in undifferentiated hTERT-FPC, whereas hTERT-FPC-Pdx1 cells cultured without serum and with activin A contained 5 ± 3 ng insulin per 1 × 10^6 ^cells. This indicated that Pdx1 restored endocrine progenitor phenotype in hTERT-FPC, although activin A was required. Detailed studies of regulated insulin secretion were not attempted in view of relatively limited insulin expression in hTERT-FPC-Pdx1 cells and requirement of further differentiation in cells.

### Endodermal-mesenchymal phenotype conversions

**Figure 6:**
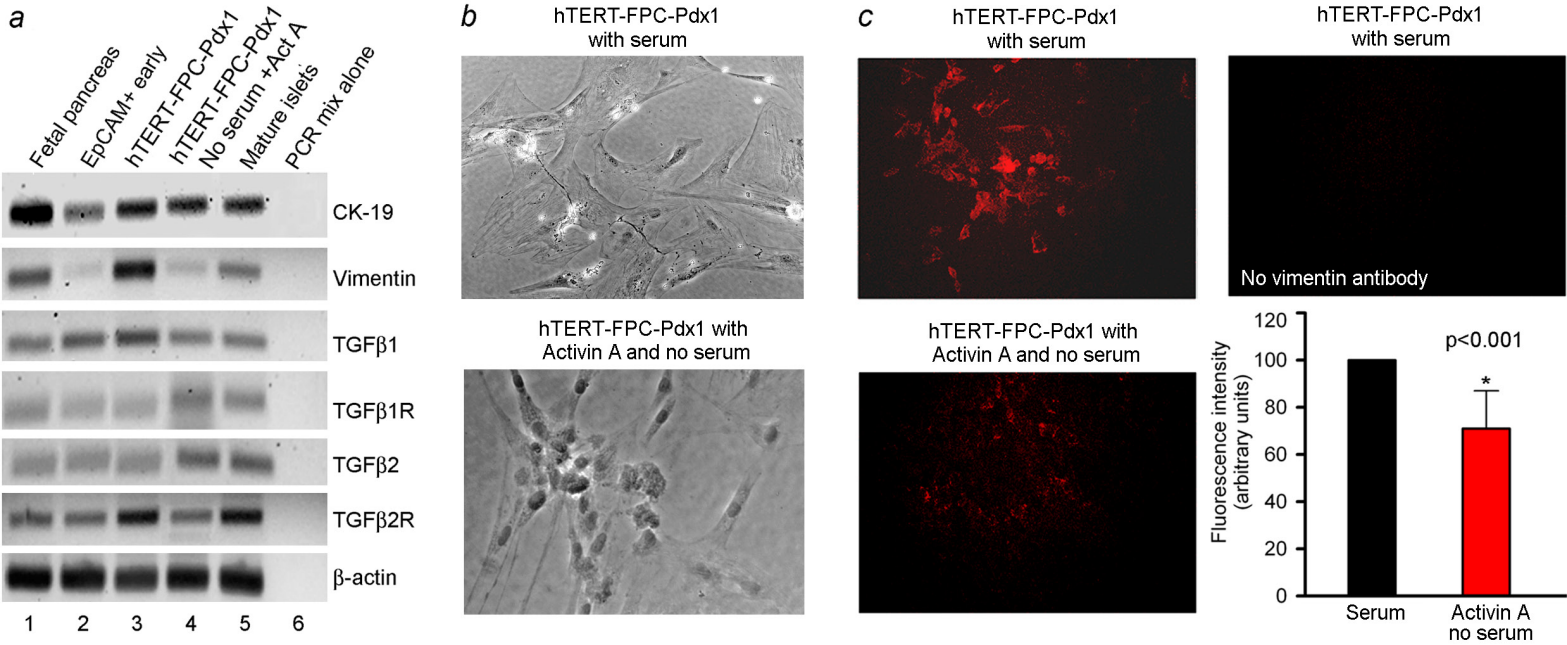
**Phenotype alterations in fetal pancreatic cells**. **(a) **shows RT-PCR for epithelial marker, CK-19, and mesenchymal marker, vimentin, along with TGF-β1, TGFβ2 and their receptors under various conditions indicated. **(b) **shows morphological changes in LV-Pdx1-transduced hTERT-FPC during culture with serum and in the absence of serum plus addition of Activin A (bottom panel). These data indicated that cells became more rounded and less flattened in the absence of serum and presence of Activin A. **(c) **shows changes in vimentin expression by immunostaining in LV-Pdx1-transduced hTERT-FPC cultured with serum (top left), and with Activin A and no serum (bottom left). No immunostaining was detected when vimentin antibody was omitted (top right). The panel at bottom right in c shows quantitation of vimentin immunofluorescence signals by image analysis to indicate that culture without serum and with activin A perturbed cell phenotype, which was in agreement with morphological changes in LV-Pdx1-transduced hTERT-FPC.

To examine the phenotype-regulating mechanism of activin A, we determined whether hTERT-FPC transitioned to an epithelial-mesenchymal state, which was recently recognized in endodermal cells [9]. We found that EpCAM-positive fetal pancreatic cells expressed the intermediate filament characteristic of epithelial cells, cytokeratin (CK)-19, with minimal expression of the mesenchymal filament, vimentin (Figure 6a). By contrast, in Pdx1-LV-transduced hTERT-FPC, expression of vimentin increased, while CK-19 was also expressed, indicating a mixed epithelial-mesenchymal phenotype. After activin A, expression of vimentin declined without affecting CK-19 expression. It was noteworthy that expression of TGF-β1, TGF-β2 and their receptors was unchanged. This was associated with changes in cell morphology, such that cultured hTERT-FPC were larger and flatter in the presence of serum, whereas hTERT-FPC-Pdx1 cells became smaller and rounder when cultured without serum and in the presence of activin A (Figure 6b). Moreover, these morphological changes were accompanied by decreased expression of vimentin, which was verified by immunostaining of cultured cells (Figure 6c).

### Transplantation of hTERT-FPC cells in NOD/SCID mice

**Figure 7:**
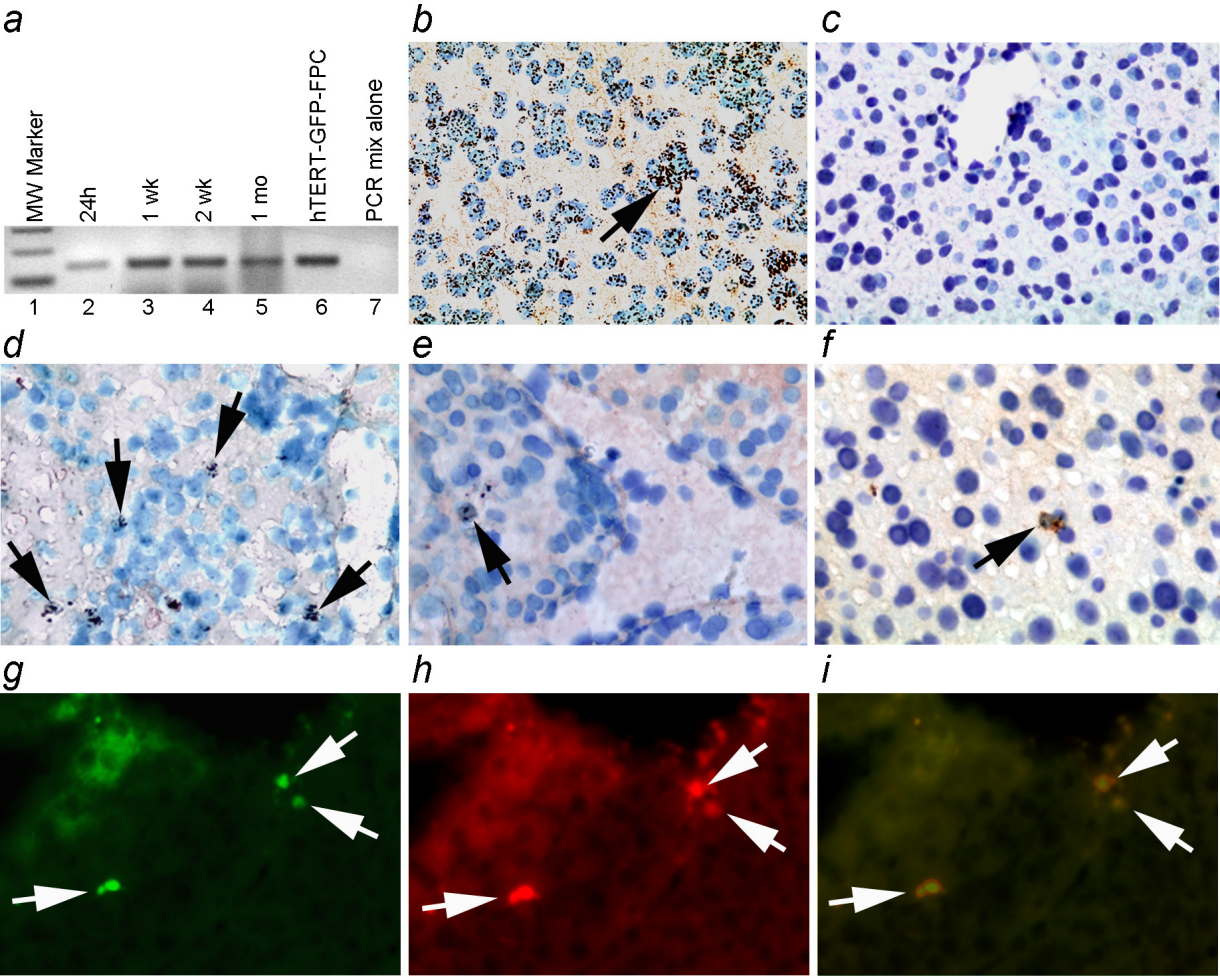
**Transplantation studies with hTERT-FPC**. **(a) **shows DNA PCR for human sequences to identify hTERT-FPC in the liver of NOD/SCID mice 24 hours, 1 week, 2 weeks and 1 month after intrasplenic transplantation. **(b-f) **show in situ hybridization for alphoid satellite sequences in human centromeres to verify that transplanted cells were present in tissues. (b), fetal human liver as positive control to show hybridization signals in cell nuclei (arrow); (c), mouse liver showing absence of hybridization signals; (d), (e) and (f) show tissues from animals 24 hours, 1 week and 1 month after cell transplantation, respectively, with transplanted cells localized by nuclear in situ signals (arrows). **(g-i) **show sequential immunostaining for GFP (g) and insulin (h) with merged image of these two panels (i) two weeks after transplantation in the liver to verify β cell phenotype in transplanted hTERT-FPC.

The fate of hTERT-FPC was established in vivo by transplantation studies in mice. Transplanted hTERT-FPC were present in the liver throughout the four-week period of studies (Figure 7a). Transplanted cells were localized in the liver by in situ hybridization (Figure 7b and 7f). Moreover, presence of GFP expression, as well as simultaneous expression of insulin in GFP-positive hTERT-FPC that had been modified by Pdx1-LV was observed (Figure 7g and 7i). No tumors were detected in animals after cell transplantation over the four-week period of the study.

## Discussion

We successfully isolated epithelial progenitor cells from the fetal human pancreas and isolated cells were greatly expanded after genetic modification with hTERT in vitro. These cells exhibited endocrine progenitor phenotype with multiple pancreatic hormones, although this phenotype was altered in culture conditions with acquisition of additional mesenchymal/mesodermal properties. As this phenotype change occurred through spontaneous processes, we believe the availability of hTERT-modified pancreatic cells will offer additional substrates for dissecting regulatory mechanisms in beta cell differentiation. In particular, our findings should be of interest in determining whether other stem/progenitor cells will transition through similar fetal stages during generation of β or beta-like cells.

During development, pancreatic progenitor cells arise from the primitive foregut endoderm, with cell-specification requiring Pdx1 and other transcription factors, for example, HNF homeobox B, NGN3, pancreas-specific transcription factor 1a, GATA4, and HNF6 [12]. We were able to isolate pancreatic epithelial progenitor cells characterized by the epithelial-specific adhesion molecule, EpCAM, which is a feature of endodermal stem/progenitor cells [8,9], although these cells demonstrated limited proliferation capacity in culture conditions. The replication potential of fetal human liver stem/progenitor cells expressing EpCAM was similarly limited and was accompanied by telomere shortening, which could be circumvented by hTERT expression [10]. In the same way, modification of pancreatic progenitor cells with hTERT greatly expanded their capacity to proliferate. The ability of other somatic cells, and even iPS cells, to assume greater proliferation capacity after modification with hTERT has been established [6,10]. This role of hTERT should be noteworthy because of the absence of tumorigenicity in hTERT-modified cells [10,20], as shown previously, and again in our cell transplantation studies here, where transplanted cells did not generate foci of proliferating cells and no tumors were observed over at least four weeks.

We do not propose that genetically-modified cells with hTERT expression will be appropriate for immediate clinical applications, as more information will be needed in regards to their biological properties, regulation of differentiation through nonintegrating vectors in case of gene therapy-type approaches or of alternative small molecule approaches for this purpose, as recently identified during β lineage derivation in hESC [21]. The availability of fetal cells described here will permit comparative studies of differentiation mechanisms in pluripotent stem cells versus stem/progenitor cells with specific lineage commitment. Our preliminary studies indicated that (-)-indolactam V, which was effective for inducing beta cell maturation in hESC-derived cells [21], failed to enhance beta cell phenotype in hTERT-FPC, suggesting fundamental differences in regulation of beta cell differentiation in various types of stem/progenitor cells.

The loss of insulin expression in cultured FPC was in agreement with previous studies, for example, downregulation of hormone expression was a feature of immortalized human pancreatic cell lines, as well as of growth-stimulated primary human pancreatic cells [22,23]. Whether loss of gene expression was due to transcriptional downregulation, independent of cell differentiation, or switching of progenitor cells to more primitive states, were both possibilities. In our studies, EpCAM-positive fetal epithelial cells displayed properties of islet progenitor cells with expression of multiple hormones and relevant transcription factors in early cell culture conditions. We found that loss of insulin expression was coupled with simultaneous loss of Pdx1 expression in hTERT-FPC. Subsequently, we found that although this initial epithelial/endodermal phenotype was maintained in cells, additional properties typically encountered in mesenchymal/mesodermal cells, for example, vimentin expression, were displayed. This was strongly reminiscent of changes in EpCAM-positive fetal human liver stem/progenitor cells, where downregulation of the epithelial/endodermal phenotype was due to the onset of conjoint meso-endoderm (epithelial plus mesenchymal) phenotype under culture conditions [9]. Also, these fetal human liver cells regained expression of epithelial genes after culture under serum-free conditions, which strengthened sharing of mechanisms observed in fetal pancreatic cells here.

Therefore, our findings of increased insulin expression in hTERT-FPC following culture under serum-free conditions with activin A were in agreement with the role of pathway-specific soluble signals in cell differentiation. The role of activin A in enhancing insulin expression in hTERT-FPC resembled the role of inductive signaling in generating β-like cells from hESC [13], and of extrinsic signaling in inducing hepatic properties in fetal stem/progenitor cells [9]. These shared mechanisms will offer new opportunities for exploring genetic and epigenetic processes in pancreatic lineage regulation, including induction of the beta cell phenotype. The availability of hTERT-FPC and hTERT-FH-B cells derived from fetal liver and pancreas, respectively, should provide important opportunities for comparing hESC- or iPS-derived cells in understanding transitions of embryonic-to-fetal and fetal-to-adult stages in the β cell lineage. For instance, it should be relevant to determine whether candidate approaches capable of advancing β cell maturation in hESC-derived cells, could be equally effective in hTERT-FPC or hTERT-FH-B cells.

As hTERT-FPC were capable of expressing insulin in vivo, this was similar to the expression of hepatic functions in fetal human liver epithelial cells following transplantation in xenotolerant mice [9]. In other studies, transplantation of hTERT-FH-B fetal liver cells modified by Pdx1, was successful in correcting hyperglycemia in mice [11,24]. Here, our cell transplantation studies were limited to establishing differentiation of hTERT-FPC in the liver. Whether these cells could correct hyperglycemia in animals will require detailed studies in the future, particularly after further differentiation and maturation of cells along the β lineage.

## Conclusions

Insights into lineage-switching mechanisms during expansion of pancreatic stem/progenitor cells will be critical for understanding the fundamental nature of expanded cells. The availability of hTERT-FPC should facilitate efforts to identify effective mechanisms to maintain stem/progenitor cells under suitable states for cell expansion without extinction of differentiation capacity. This will be useful for translational applications, including cell therapy in type-1 diabetes mellitus.

## Abbreviations

BETA2: betacellulin 2; BSA: bovine serum albumin; CaCl_2_: calcium chloride; cDNA: complementary deoxyribonucleic acid; CGA: chromogranin A; CK: cytokeratin; CMT: Charcot-Marie-Tooth; DMEM: Dulbecco's Minimal Eagle Medium; Dnase: deoxyribosenuclease; DPPIV: dipeptidyl peptidase IV; EDTA: ethylenediaminetetraacetic acid; EpCAM: epithelial cell adhesion molecule; FBS: fetal bovine serum; FITC: fluorescein isothiocyanate; GATA: GATA family of transcription factors; GFP: green fluorescent protein; GGT: γ-glutamyltranspeptidase; GK: glucokinase; GLUT: glucose transporter; HEPES: 4-(2-hydroxyethyl)-1-piperazineethanesulfonic acid; hESC: human embryonic stem cells; HNF: hepatic nuclear factor; hTERT: human telomerase reverse transcriptase; hTERT-FPC: human telomerase reverse transcriptase containing fetal pancreatic cells; IGF-1R: insulin-like growth factor-1 receptor; iPS: inducible pluripotential stem cells; Isl: islet; KCl:: potassium chloride; LV: lentivirus vector; NaCl: sodium chloride; NaH_2_PO_4_: sodium dihydrogen phosphate; NGN: Neurogenin; Nkx: NK homeobox protein; NOD-SCID: natural onset diabetes-severe combined immunodeficiency; Pax: paired homeobox gene; PBS: phosphate buffered saline; PC: prohormone convertase; pCCLsinPPT.hPGK.IRES.GFP.Wpre: third generation lentivirus plasmid; Pdx: pancreatic duodenal homeobox gene; PGK: phosphogylcerate kinase; pMD2.G: plasmid construct expressing vesicular stomatitis virus G envelope protein; pMDLg/pRRE: plasmid containing human immunodeficiency virus-derived packaging construct; PP: pancreatic polypeptide; pRSV-Rev: plasmid construct expressing Rev regulatory protein; RT-PCR: reverse-transcription polymerase chain reaction; TGF: transforming growth factor.

## Competing interests

The authors declare that they have no competing interests.

## Authors' contributions

KC performed studies, interpreted data, and prepared the manuscript. AF performed studies, interpreted data, and reviewed the manuscript. MS performed studies, interpreted data, and reviewed the manuscript. NF interpreted data, reviewed and approved the manuscript. SG designed studies, interpreted data and prepared the manuscript.

## References

[B1] Hao E Tyrberg B Itkin-Ansari P Lakey JR Geron I Monosov EZ Barcova M Mercola M Levine F Beta-cell differentiation from nonendocrine epithelial cells of the adult human pancreas Nat Med 2006 12 310 316 10.1038/nm1367 16491084

[B2] Xu X D'Hoker J Stangé G Bonné S De Leu N Xiao X Casteele M Van de Mellitzer G Ling Z Pipeleers D Bouwens L Scharfmann R Gradwohl G Heimberg H β Cells Can Be Generated from Endogenous Progenitors in Injured Adult Mouse Pancreas Cell 2008 132 197 207 10.1016/j.cell.2007.12.015 18243096

[B3] Halvorsen TL Beattie GM Lopez AD Hayek A Levine F Accelerated telomere shortening and senescence in human pancreatic islet cells stimulated to divide in vitro J Endocrinol 2000 166 103 109 10.1677/joe.0.1660103 10856888

[B4] Fleischer N Chen C Surana M Leiser M Rossetti L Pralong W Efrat S Functional analysis of a conditionally transformed pancreatic beta-cell line Diabetes 1998 47 1419 1425 10.2337/diabetes.47.9.1419 9726230

[B5] Wang S Beattie GM Mally MI Cirulli V Itlin-Ansari P Lopez AD Hayek A Levine F Isolation and characterization of a cell line from the epithelial cells of the human fetal pancreas Cell Transplant 1997 6 59 67 10.1016/S0963-6897(96)00120-0 9040956

[B6] Park IH Zhao R West JA Yabuuchi A Huo H Ince TA Lerou PH Lensch MW Daley GQ Reprogramming of human somatic cells to pluripotency with defined factors Nature 2008 451 141 146 10.1038/nature06534 18157115

[B7] Zhou Q Brown J Kanarek A Rajagopal J Melton DA In vivo reprogramming of adult pancreatic exocrine cells to beta-cells Nature 2008 455 627 632 10.1038/nature07314 18754011 PMC9011918

[B8] Inada M Benten D Cheng K Joseph B Berishvili E Badve S Logdberg L Dabeva M Gupta S Stage-specific regulation of adhesion molecule expression segregates epithelial stem/progenitor cells in fetal and adult human livers Hepatol Int 2008 2 50 62 10.1007/s12072-007-9023-4 19669279 PMC2716863

[B9] Inada M Follenzi A Cheng K Surana M Joseph B Benten D Bandi S Qian H Gupta S Phenotype reversion in fetal human liver epithelial cells identifies the role of an intermediate meso-endodermal stage before hepatic maturation J Cell Sci 2008 121 1002 1013 10.1242/jcs.019315 18319302 PMC2695499

[B10] Wege H Le HT Chui MS Liu L Wu J Giri RK Malhi H Sappal BS Kumaran V Gupta S Zern MA Telomerase reconstitution immortalizes human fetal hepatocytes without disrupting their differentiation potential Gastroenterology 2003 124 432 444 10.1053/gast.2003.50064 12557149

[B11] Zalzman M Gupta S Giri RK Berkovich I Sappal BS Karnieli O Zern MA Fleischer N Efrat S Reversal of hyperglycemia in mice using human expandable insulin-producing cells differentiated from fetal liver progenitor cells Proc Natl Acad Sci USA 2003 100 7253 7258 10.1073/pnas.1136854100 12756298 PMC165862

[B12] Zaret KS Genetic programming of liver and pancreas progenitors: lessons for stem-cell differentiation Nat Rev Genet 2008 9 329 340 10.1038/nrg2318 18398419

[B13] Shiraki N Yoshida T Araki K Umezawa A Higuchi Y Goto H Kume K Kume S Guided differentiation of embryonic stem cells into Pdx1-expressing regional-specific definitive endoderm Stem Cells 2008 26 874 885 10.1634/stemcells.2007-0608 18238854

[B14] Follenzi A Ailles LE Bakovic S Geuna M Naldini L Gene transfer by lentiviral vectors is limited by nuclear translocation and rescued by HIV-1 pol sequences Nat Genet 2000 25 217 222 10.1038/76095 10835641

[B15] Heremans Y Casteele M Van De in't Veld P Gradwohl G Serup P Madsen O Pipeleers D Heimberg H Recapitulation of embryonic neuroendocrine differentiation in adult human pancreatic duct cells expressing neurogenin 3 J Cell Biol 2002 159 303 312 10.1083/jcb.200203074 12403815 PMC2173047

[B16] Schwartz RE Reyes M Koodie L Jiang Y Blackstad M Lund T Lenvik T Johnson S Hu WS Verfaillie CM Multipotent adult progenitor cells from bone marrow differentiate into functional hepatocyte-like cells J Clin Invest 2002 109 1291 1302 12021244 10.1172/JCI15182 PMC150983

[B17] Piao CQ Liu L Zhao YL Balajee AS Suzuki M Hei TK Immortalization of human small airway epithelial cells by ectopic expression of telomerase Carcinogenesis 2005 26 725 731 10.1093/carcin/bgi016 15677631

[B18] Benten D Follenzi A Bhargava KK Kumaran V Palestro CJ Gupta S Hepatic targeting of transplanted liver sinusoidal endothelial cells in intact mice Hepatology 2005 42 140 148 10.1002/hep.20746 15918158

[B19] Benten D Cheng K Gupta S Identification of transplanted human cells in animal tissues Methods Mol Biol 2006 326 189 201 16780202 10.1385/1-59745-007-3:189

[B20] Jiang XR Jimenez G Chang E Frolkis M Kusler B Sage M Beeche M Bodnar AG Wahl GM Tlsty TD Chiu CP Telomerase expression in human somatic cells does not induce changes associated with a transformed phenotype Nat Genet 1999 21 111 114 10.1038/5056 9916802

[B21] Chen S Borowiak M Fox JL Maehr R Osafune K Davidow L Lam K Peng LF Schreiber SL Rubin LL Melton D A small molecule that directs differentiation of human ESCs into the pancreatic lineage Nat Chem Biol 2009 5 258 265 10.1038/nchembio.154 19287398

[B22] Itkin-Ansari P Demeterco C Bossie S Tour DD Beattie GM Movassat J Movassat J Mally MI Hayek A Levine F Pdx1 and cell-cell contact act in synergy to promote δ-cell development in a human pancreatic endocrine precursor cell line Mol Endocrinol 2000 14 814 822 10.1210/me.14.6.814 10847584

[B23] Ramiya VK Maraist M Arfors KE Schatz DA Peck AB Cornelius JG Reversal of insulin-dependent diabetes using islets generated in vitro from pancreatic stem cells Nat Med 2000 6 278 282 10.1038/73128 10700229

[B24] Zalzman M Anker-Kitai L Efrat S Differentiation of human liver-derived, insulin-producing cells toward the beta-cell phenotype Diabetes 2005 54 2568 2575 10.2337/diabetes.54.9.2568 16123344

