# Erratum to: Switching of mesodermal and endodermal properties in hTERT-modified and expanded fetal human pancreatic progenitor cells

**DOI:** 10.1186/s13287-015-0176-0

**Published:** 2015-09-30

**Authors:** Kang Cheng, Antonia Follenzi, Manju Surana, Norman Fleischer, Sanjeev Gupta

**Affiliations:** Hepatology Division, Department of Medicine, Albert Einstein College of Medicine, Ullmann Bldg., Rm 625, 1300 Morris Park Avenue, Bronx, NY 10461 USA; Department of Pathology, Albert Einstein College of Medicine, Ullmann Bldg., Rm 625, 1300 Morris Park Avenue, Bronx, NY 10461 USA; Endocrinology Division, Department of Medicine, Diabetes Research Center, Albert Einstein College of Medicine, Forchheimer Bldg., Rm 505, 1300 Morris Park Avenue, Bronx, NY 10461 USA; Hepatology Division, Department of Medicine, Cancer Research Center, Diabetes Research Center, Center for Human Embryonic Stem Cell Research, Marion Bessin Liver Research Center, Ruth L. and David S. Gottesman Institute for Stem Cell and Regenerative Medicine Research, and Institute for Clinical and Translational Research, Albert Einstein College of Medicine, Ullmann Bldg., Rm 625, 1300 Morris Park Avenue, Bronx, NY 10461 USA

## Erratum

After publication of our article [1], errors were noticed in the composition of data in Figures threeC, fiveD and sixA (Figs. 1c, 2d and 3a here respectively). The data from original gels were incorrectly compiled or modified by the first author, Dr. K. Cheng, which was not noticed by the other authors.

**Fig. 1:**
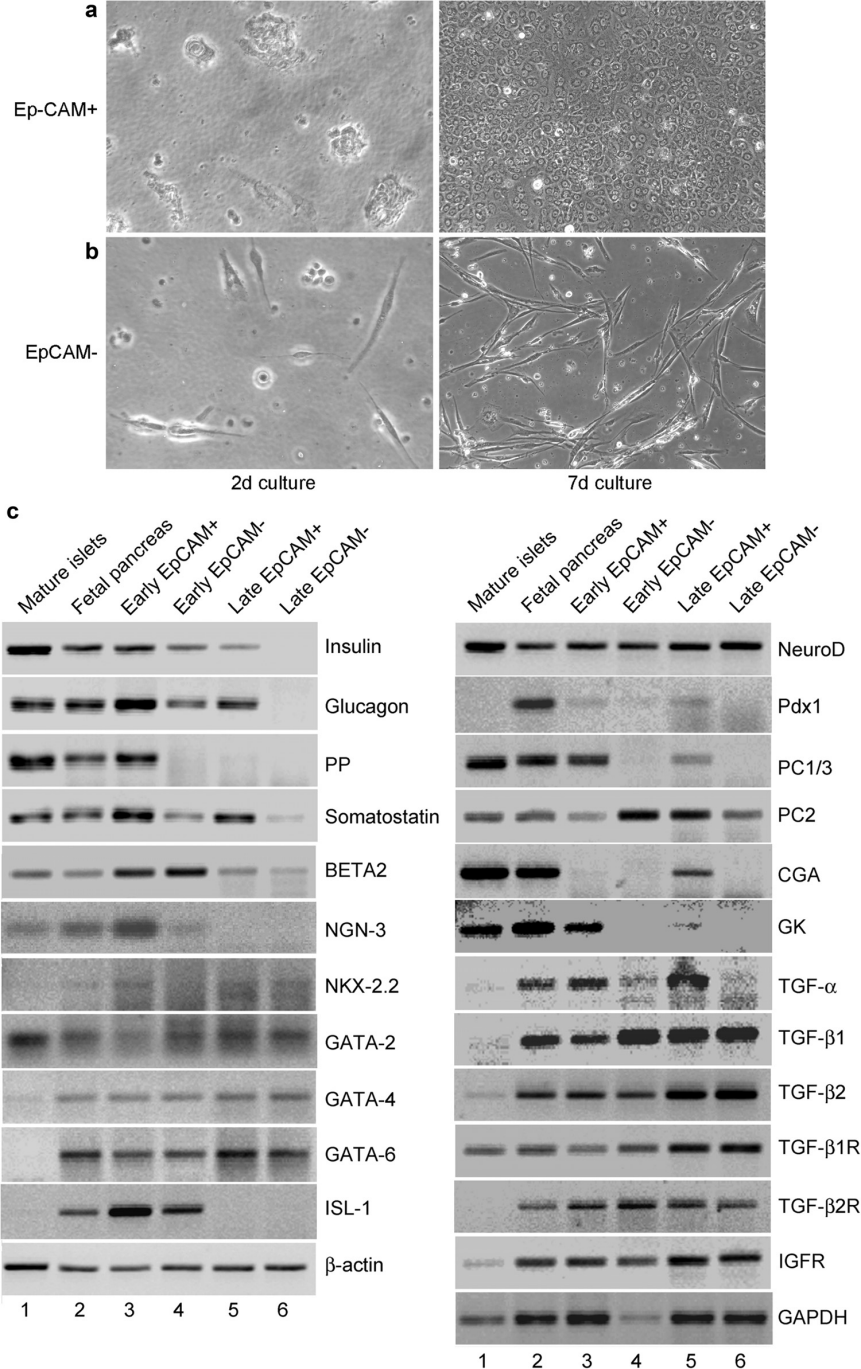
Initial characterization of fetal pancreatic cells. **a** and **b** show morphology of cells in culture after 2 d and 7 d. Note epithelial morphology of EpCAM-positive cells. **c** shows RT-PCR for genes as indicated. Lanes 1 to 6 show results from mature human pancreatic islets, intact fetal pancreas, cells after early term culture (1 to 2 d) or longer culture (10 to 14 d). For comparisons, β-actin and glyceraldehyde phosphate dehydrogenase (GAPDH) genes were included

**Fig. 2:**
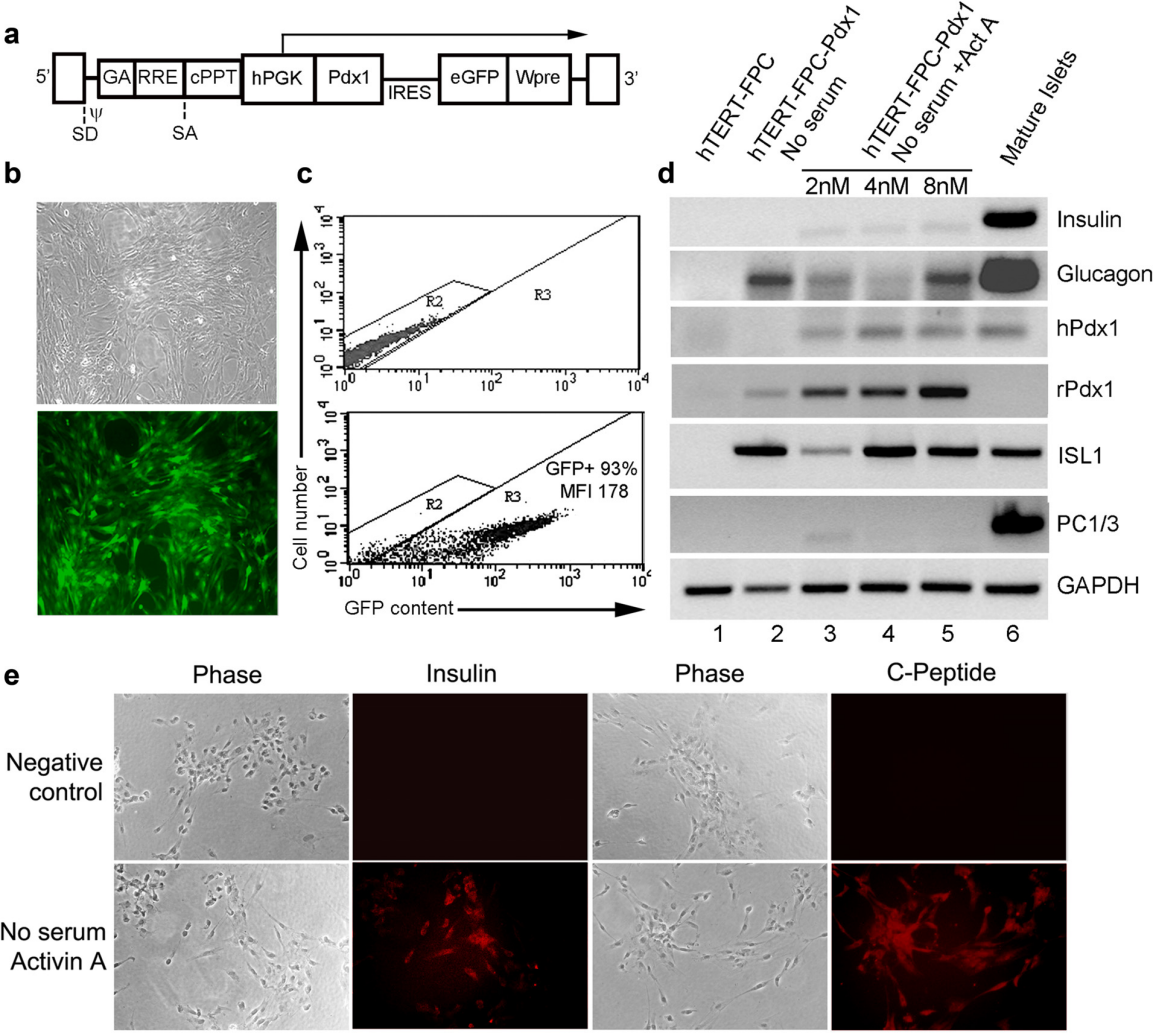
Induction of insulin-expression in hTERT-FPC by Pdx1-LV. **a** shows schematic of LV with rat Pdx1 and GFP genes driven by hPGK promoter - IRES, intervening internal ribosomal entry site, cPPT, central polypurine tract, Wpre, posttranscriptional regulatory element of the woodchuck hepadnavirus. **b** shows Pdx1-LV-transduced hTERT-FPC under phase contrast (top) and under epifluorescence for GFP. **c** shows flow cytometric quantitation of GFP in nontransduced cells (top panel) and Pdx1-LV-transduced hTERT-FPC. MFI = mean fluorescence intensity. **d** shows RT-PCR for gene expression in control hTERT-FPC (lane 1), Pdx1-LV-transduced hTERT-FPC cultured without serum (lane 2) and without serum plus activin A (lane 3), and mature pancreatic islets (lane 4). **e** shows insulin and c-peptide expression in negative control hTERT-FPC-Pdx1 cells, where primary antibodies were omitted, and cells with expression of both insulin and c-peptide. Orig. Mag., × 200

**Fig. 3:**
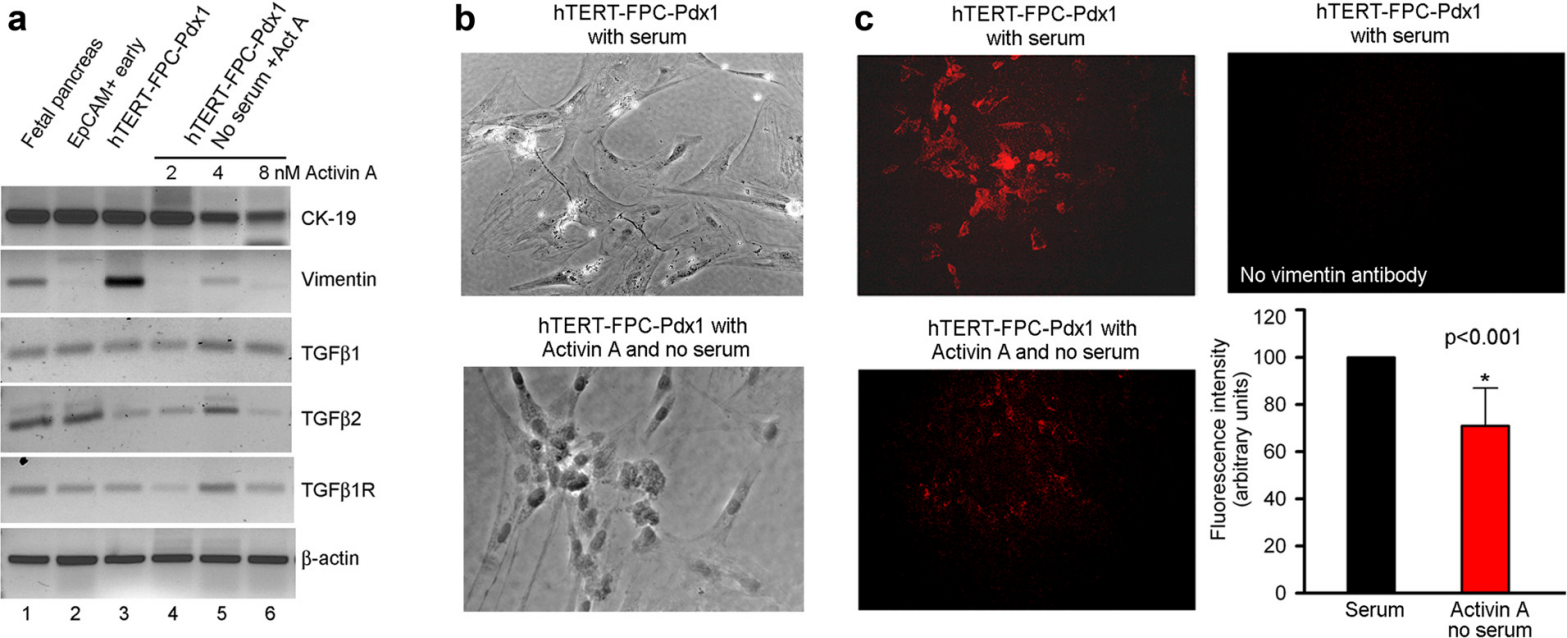
Phenotype alterations in fetal pancreatic cells. **a** shows RT-PCR for epithelial marker, CK-19, and mesenchymal marker, vimentin, along with TGF-β1, TGFβ2 and their receptors under various conditions indicated. **b** shows morphological changes in LV-Pdx1-transduced hTERT-FPC during culture with serum and in the absence of serum plus addition of Activin A (bottom panel). These data indicated that cells became more rounded and less flattened in the absence of serum and presence of Activin A. **c** shows changes in vimentin expression by immunostaining in LV-Pdx1-transduced hTERT-FPC cultured with serum (top left), and with Activin A and no serum (bottom left). No immunostaining was detected when vimentin antibody was omitted (top right). The panel at bottom right in c shows quantitation of vimentin immunofluorescence signals by image analysis to indicate that culture without serum and with activin A perturbed cell phenotype, which was in agreement with morphological changes in LV-Pdx1-transduced hTERT-FPC

In figure threeC (Fig. 1c here) the original gels for the following genes were incorrectly represented – GATA-2, GATA-6, ISL-1, Pdx1, CGA, GK, TGF-α, TGF-β1, TGF-β2, TGF-β2R, and GAPDH. Expression of these genes in mature islets was verified by additional studies.

In figure fiveD (Fig. 2d here) some of the lanes were cut out of the composition, and others were mislabeled.

In figure sixA (Fig. 3a here) the published figure was erroneously composed with incorrect or distorted images.

The correct figures are provided here. These errors do not affect the results or conclusions of our study.

Please note the change in corresponding author email address since the publication of our original article.
